# Applying the Multiphase Optimization Strategy for the Development of a Culturally Tailored Resilience-Building Intervention to Facilitate Advance Care Planning Discussions for Chinese Americans: Protocol for a Survey and Qualitative Study

**DOI:** 10.2196/59343

**Published:** 2024-11-26

**Authors:** Li-Ting Huang Longcoy, Chien-Ching Li, Chun-Yi Tai, Ardith Doorenbos

**Affiliations:** 1 College of Nursing University of Illinois Chicago Chicago, IL United States; 2 Department of Health Systems Management Rush University Chicago, IL United States; 3 School of Nursing National Taipei University of Nursing and Health Sciences Taipei Taiwan

**Keywords:** resilience, Chinese Americans, multiphase optimization strategy, protocol, advance care planning, feasibility studies

## Abstract

**Background:**

Newly arrived Chinese Americans face difficulties engaging in advance care planning (ACP) discussions with their family caregivers. Avoiding such discussions and failing to complete advance directives can delay palliative and hospice care. Yet, timely palliative care is essential to maintaining the quality of life at the end of life. Currently, there is a lack of interventions to help Chinese Americans diagnosed with cancer or heart disease overcome the barriers to engaging in ACP discussions via effective use of resilience.

**Objective:**

This study aims to develop a culturally tailored, resilience-building intervention for Chinese Americans with cancer or heart disease.

**Methods:**

The development of this intervention will be guided by the 3-phase multiphase optimization strategy. In the first phase of preparation, we will examine the prespecified components of the intervention through pilot studies to understand the necessity of each component. First, a qualitative study will be conducted to understand the experiences of 10 religious or spiritual leaders who have provided pastoral or spiritual care to Chinese Americans in Chicago, United States. The interview findings will be categorized as facilitators and barriers and integrated into the development of the intervention’s resilience-building guide. Second, a cross-sectional study will be conducted to assess the readiness of Chinese Americans to engage in ACP discussions with their family using surveys. Third, think-aloud interviews will be conducted to understand the experiences of 18 pairs of Chinese Americans and their family caregivers regarding the prototype of the culturally tailored, resilience-building intervention. Finally, we will examine the feasibility and acceptability of the intervention prototype along with issues related to the study’s implementation process.

**Results:**

Recruitment for the qualitative study began in November 2023. As of October 2024, a total of 7 participants have been recruited, enabling a preliminary qualitative analysis to evaluate the analytical framework developed from the literature. Recruitment for the cross-sectional study began in April 2024, and as of October 2024, a total of 63 Chinese Americans have participated. The potential participant recruitment lists for the think-aloud interviews have been received, enabling recruitment to begin after the preliminary qualitative analysis is completed.

**Conclusions:**

The proposed culturally tailored, resilience-building intervention is an innovative way to facilitate ACP discussions among Chinese Americans, particularly those diagnosed with serious chronic diseases. The findings from all 3 study methods will inform the development of the proposed intervention and identify effective recruitment strategies for this underserved and hard-to-reach population to be used in future research.

**International Registered Report Identifier (IRRID):**

DERR1-10.2196/59343

## Introduction

Advance care planning (ACP) is an iterative process that helps adults reflect on their personal values and preferences regarding future medical care and document those preferences in advance directives to share with their health care agents and health care providers [1]. ACP encompasses 3 main components, including designating a health care agent, discussing ACP with the health care agent, and completing an advance directive with the health care agent [2]. The well-known benefits of ACP include its ability to (1) help individuals increase satisfaction with care, patient-family communication, and quality of death; and (2) improve the mental health of family caregivers by reducing anxiety, depression, and caregiver decisional burden [3]. Despite the proven benefits of ACP, however, only 13.7% of Chinese Americans (defined for this study as individuals who self-identify as of Chinese descent, including foreign-born immigrants from regions such as China, Hong Kong, Taiwan, and other East Asian countries, as well as the descendants of these first-generation immigrants; hereafter referred to as Chinese Americans) have completed advance directives [4]. This rate is less than half of the 37% completion rate in the US general population [5]. This disparity in the use of ACP between Chinese Americans and non-Hispanic White Americans (defined as US residents with origins in Europe, the Middle East, or North Africa) [6] may relate to known disparities between these groups in palliative and end-of-life care, including in rates of hospice care use, the prevalence of unwanted aggressive treatments at the end of life, and end-of-life expenditures [7-10]. For example, Chinese Americans are twice as likely as White Americans to be administered mechanical ventilation in their last 30 days of life (17% compared to 8%) and significantly more likely to die in a hospital (49% compared to 36%) [9]. In addition, the mean end-of-life Medicare expenditures claimed by Asian Americans (US $15,388) are significantly higher than those claimed by White Americans (US $10,761) [10]. In other words, the lack of completion of advance directives among Chinese Americans may contribute to aggressive and extravagant care that significantly worsens their quality of life at the end of their lives.

The leading causes of death among Chinese Americans—similar to those for non-Hispanic White Americans—are cancer and heart disease [11]. Yet, newly arrived Chinese Americans’ health behaviors regarding access to care, specifically end-of-life care, may contribute to health disparities at the end of life. When Chinese Americans face a diagnosis of serious chronic diseases, such as cancer or heart disease, their coping behaviors are deeply affected by four cultural beliefs: (1) fatalism; (2) respect for elders; (3) harmony; and (4) respect for authority figures, such as health care providers and religious leaders [12].

Chinese Americans may hold the fatalistic belief that life is predetermined by destiny and beyond one’s control [12]. This fatalistic belief may reduce Chinese Americans’ willingness to adopt healthy behaviors, including ACP discussions and prevention-focused cancer screening [12-14]. Some may even deny their cancer diagnosis as a coping mechanism [15], and others may turn to religious or spiritual practices as a way to cope with serious disease [16]. Their family caregivers, a role usually served by adult children, may show respect for elders by protecting and promoting their parents’ well-being [12]. However, when adult children know that taboo topics like ACP may offend their parents, they may avoid these uncomfortable conversations, even if their parents initiate them [17]. To maintain family harmony, adult children may wait for authority figures, especially health care providers, to initiate ACP discussions [17]. In situations where ACP topics have not been initiated by health care providers, adult children who practice filial piety (ie, the obligation felt by children to ensure the well-being of their parents in order to promote longevity) may decide to prolong their parents’ lives by selecting life-sustaining therapy during end-of-life care decision-making [12,18]. However, this decision, despite being based on goodwill, may contradict the Chinese cultural value related to a good death, in which being pain-free and maintaining dignity in death are preferred [12,19]. These cultural beliefs, combined with other common barriers, including misconceptions about ACP [20] and low levels of acculturation and health literacy [21,22], may exacerbate the low completion rate of advance directives among Chinese Americans.

Making end-of-life decisions requires constant coping with barriers while considering cultural beliefs. One promising approach to helping Chinese Americans appraise and cope with the barriers to ACP is building resilience. In this study, we define resilience as the process by which an individual learns to use their resources to sustain physical and psychological well-being in the face of adversity [23,24]. Resilience may enable individuals to buffer against stress, and it can be developed through practice [25]. Individuals with resilience skills can effectively use and apply their external resources (eg, social support from families and health care providers), internal resources (eg, individual strengths and coping skills), and existential resources (eg, meaning-making and finding gratitude) to address the stress they face [26]. Individuals with resilience skills may see a serious illness diagnosis as a challenge instead of a threat and use suitable coping strategies such as self-awareness of personal strengths and cultivating positive emotions and positive relationships with health care agents to facilitate psychological adaptation [27]. Continuous adaptation, through accurate appraisals and evaluation of coping strategies, is an important process for building the resilience necessary to overcome the stress experienced in ACP discussions.

Previous clinical trials of interventions have consistently shown positive effects of resilience on reducing psychological distress, increasing personal growth, and improving the quality of life in people with cancer [28,29]. Patients with breast cancer who participated in a pilot randomized controlled trial (RCT) showed improved resilience and decreased anxiety and perceived stress after 12 weeks of resilience training, including training in finding gratitude, meaning-making, and interpreting the cancer experience flexibly [28]. Family caregivers of patients with cancer can also benefit from the protective effects of resilience. One study found resilience to be negatively associated with caregiving burden, anxiety, and depression among family caregivers of patients with advanced cancer [30]. Resilience was also found to be a predictor of a caregiver’s readiness for surrogate decision-making [31]. These studies suggest that strengthening the resilience of people with cancer or heart disease and their family caregivers may help reduce the emotional impact of ACP discussions and improve the process of completing advance directives.

Currently, most ACP interventions developed for Chinese Americans in the United States have been examined for effectiveness at the patient level only [32-34], overlooking the collective family decision-making process emphasized in Chinese culture and the ongoing evaluations needed to stay current with evolving preferences and values for end-of-life care [22]. In addition, despite the need for spiritual support, especially during difficult times [16], very few studies have included religious leaders in the development of interventions to understand how they address the barriers faced by Chinese Americans regarding ACP and death-related issues. Additionally, most prior interventions examined changes in knowledge and attitudes among community-dwelling Chinese Americans [32-34], not those currently experiencing serious chronic diseases such as cancer or heart disease. While these interventions have demonstrated improvements in knowledge and attitudes toward ACP [32], they may not adequately address the evolving preferences and values regarding end-of-life care when Chinese Americans confront their own mortality. A diagnosis of cancer or heart disease may prompt a greater need for resilience skills to successfully engage in ACP discussions with their family caregivers [13,16].

As a first-generation Chinese American nurse scientist specializing in palliative and hospice care in the United States, the first author (LL) is acutely aware of the challenges adult children face in discussing death and dying with their parents. These challenges are compounded by barriers related to health literacy and misconceptions about hospice and palliative care. The first author’s cultural background influenced her research interest in developing a culturally tailored, resilience-building intervention to help Chinese Americans engage in ACP discussions.

This feasibility study has 3 aims for intervention development, each of which uses a different study design. Aim 1 is to conduct a qualitative content analysis of semistructured interviews with 10 religious or spiritual leaders to identify barriers and facilitators to discussing ACP and death-related topics among Chinese Americans in Chicago, United States. Aim 2 is to collect survey data to assess the readiness of Chinese Americans aged 18 years and older and living in Chicago to engage in ACP discussions with their families. Aim 3 is to conduct think-aloud interviews with 18 pairs of Chinese Americans and their family caregivers (9 pairs with cancer and 9 pairs with heart disease) living in Chicago to collect feedback on the content of a culturally tailored, resilience-building intervention prototype. We expect that incorporating the strategies used by religious or spiritual leaders to discuss ACP and death-related topics (aim 1) into the intervention prototype will enhance the acceptability of the intervention among Chinese Americans and their family caregivers (as measured in aim 3). This intervention, in turn, may potentially address Chinese Americans’ readiness for ACP discussions (as identified in aim 2) and increase the completion rate of advance directives.

## Methods

### Study Design

In this study, we will use the multiphase optimization strategy (MOST) to guide the development of the culturally tailored, resilience-building intervention. MOST is an engineering-inspired framework that aims to develop a multicomponent behavioral intervention that is most efficient, affordable, and scalable [35]. It consists of 3 phases: preparation, optimization, and evaluation. In the preparation phase, intervention components are selected for inclusion and then examined through various study designs to understand the necessity of each. In the optimization phase, the final necessary components are combined and examined using a factorial experimental design to assess their individual and combined effects. In the evaluation phase, the intervention that was refined based on the results of the optimization phase is evaluated in an RCT to examine its effectiveness [36].

The proposed study will focus on the preparation phase of MOST. Based on previously reported barriers to ACP among Chinese Americans, we selected the following intervention components: (1) knowledge related to ACP [20], (2) end-of-life education [12], (3) check-ins by research staff, (4) family caregiver involvement [22], and (5) resilience skills [25]. Each component targets only one proximal mediator (Multimedia Appendix 1): (1) ACP knowledge; (2) cultural beliefs; (3) concrete support from health care providers; (4) social support from family caregivers during ACP discussions; and (5) use of adequate coping skills.

The intervention development will be informed by the interview and survey findings collected in the 3 aims. The intervention prototype will consist of five components (Multimedia Appendix 1): (1) provision of an advance directive (Five Wishes) to introduce ACP; (2) end-of-life education to overcome cultural barriers; (3) regular check-ins from a trained research staff member; (4) family caregiver involvement; and (5) a six-module resilience guide. The resilience skills taught in this intervention center around Five Wishes, a widely used advance directive that combines a living will, health care power of attorney, comfort care, and spirituality [37,38]. Patients and family caregivers will be provided with Five Wishes and the resilience guide. They will first be educated about ACP and end-of-life care, then review 1 wish from Five Wishes per module and use the resilience guide to learn 1 resilience skill to cope with that particular wish. Each module also includes a short activity to help patients and family caregivers accurately appraise the stressors and flexibly use different coping strategies for barriers related to ACP discussions.

### Ethical Considerations

This study was approved by the University of Illinois Chicago (2023-0354) and Rush University Medical Center institutional review boards (IRBs; 23061205-IRB01). Informed consent will be obtained after participants are informed that any identifiable data collected for the study will only be disclosed to others with their written permission. Participants will be compensated upon the completion of data collection (aims 1 and 3: US $50/person/hour; aim 2: US $20/person). The findings will be shared at national and regional scientific conferences, such as the American Public Health Association Annual Meeting and Society of Behavioral Medicine Annual Meeting, as well as in peer-reviewed journals and with other investigators interested in health disparities, end-of-life care, and positive psychology.

### Aim 1

#### Setting and Sample

For aim 1, we will conduct a qualitative content analysis to understand the experiences of religious or spiritual leaders and facilitate the development of the intervention prototype. The research gap in understanding ACP from the perspective of religious or spiritual leaders led to the following research question: What are the barriers and facilitators experienced by religious or spiritual leaders, including chaplains, when addressing ACP or death-related issues with Chinese Americans in Chicago? Religious or spiritual leaders play a key role as authority figures in providing spiritual care to Chinese Americans, especially following a diagnosis of serious chronic disease [16]. We will use snowball sampling to recruit 10 religious or spiritual leaders to participate in semistructured interviews, meeting the suggested minimum sample size needed to reach saturation in qualitative research [39]. Religious or spiritual leaders, regardless of their affiliation, will be eligible for inclusion in the study if they (1) are aged 18 years and older; (2) serve as a chaplain, pastor, or monk at a health care setting or religious organization in Chicago; (3) have experience providing pastoral or spiritual care to Chinese Americans living in Chicago; and (4) are able to read and respond to questions in English or Mandarin. Religious or spiritual leaders will be excluded from the study if they are not willing to provide consent.

#### Procedure

For the snowball sampling, the recruitment chain will begin with 1 chaplain and 2 pastors in the Chicago region. Considering the potential bias from snowball sampling, we will purposefully select the first 3 religious or spiritual leaders who do not have a prior relationship with the interviewer (LL). Religious or spiritual leaders will be contacted by phone or email to arrange a meeting at their preferred location and mode (either in-person or via videoconference call on Zoom [Zoom Video Communications]). During initial contact, a research team member will use a telephone script to determine if the potential participant meets the eligibility criteria. Those eligible will be given an informed consent form in either paper-and-pencil or electronic format to provide the purpose, procedures, benefits, and risks of the study. Participants will be given time to review the consent form and will be encouraged to ask questions or express any concerns about the study. In addition, they will be informed that the interview will be audio and video recorded to document their verbal and nonverbal communication, including voice, tone of voice, facial expressions, gestures, body language, and posture. After consent is obtained, participants will be asked to provide their demographic information at the start of the interview. Then, a 40- to 45-minute, one-on-one, semistructured interview will proceed to ensure a rich and detailed insight into participants’ experiences by asking the following open-ended questions: “Tell me about your end-of-life discussions with Chinese Americans living in Chicago”; “What barriers did you encounter when you addressed ACP and/or death-related topics with Chinese Americans?”; “Tell me about the strategies you used for ACP discussions and death-related issues”; and “Tell me about your experiences in providing end-of-life education to Chinese Americans.” As one of the strategies to enhance credibility, participants will be encouraged to provide examples to support their statements regarding barriers and facilitators [40]. At the end of each interview, participants will be asked to identify other religious or spiritual leaders who have similar experiences providing spiritual care to Chinese Americans in Chicago. On interview completion, each participant will receive US $50 (cash or gift card) for their time and effort. To further enhance credibility, each participant will be contacted again with an opportunity to review their own transcripts and the interpretation of the interview data and provide feedback [40]. We have already planned for the recruitment of 10 religious or spiritual leaders, with the possibility of continuing as needed until data saturation is achieved.

#### Qualitative Data Analysis

To identify barriers and facilitators of ACP, all interviews will be transcribed verbatim and entered into NVivo 14 qualitative data analysis software (Lumivero). At the beginning of the content analysis, we will use an iterative process of reading transcripts to confirm their accuracy and enhance immersion in the data [41]. The first author (LL) will analyze the qualitative data using the 6-stage framework method while developing an analytical framework. The initial analytical framework will consist of two categories: (1) barriers to death and dying discussions encountered by Chinese Americans, and (2) facilitators used by religious or spiritual leaders. Under the barriers category, predefined codes will be developed based on previous literature, including cultural beliefs, indirect communication, fear, lack of knowledge, language barriers for health care, and others. Codes under the facilitators category will be derived from the open coding process [41].

The first author will then read 2 transcripts to pilot-test the codes and revise the analytical framework should any new codes arise during this process. She will then independently read the remaining 8 transcripts and highlight all text deemed to address barriers to or facilitators of end-of-life discussions. The third author (CT) will independently code line-by-line. After the first round of coding, the authors will discuss the initial analytical framework, use interrater coding tools to compare coding differences, reconcile any disagreements, and recode the transcripts if needed. Throughout this process, we will practice reflexivity by keeping a reflexive journal, conducting peer debriefing, and engaging in critical self-reflection [42]. The first author, who will conduct all interviews, will also specifically assess whether her religious affiliation may influence the research process, including data collection and analysis.

After completing the data analysis, we will assess the necessity of recruiting additional participants based on the consensus regarding the barriers and facilitators encountered by the 10 participants. Recruitment will cease when data saturation is achieved (ie, no further addition of information) [39]. Then, we will contact the participants and share the final interpretation of data with them for clarification, feedback, and accuracy of the data interpretation, which will then be used to refine the proposed intervention. When reporting and disseminating the findings, we will use the Joanna Briggs Institute checklist for qualitative research to ensure transferability, providing a detailed description of the research process that readers will be able to apply to their own populations and settings [40].

### Aim 2

#### Setting and Sample

For aim 2, we will conduct a survey to understand the readiness of Chinese Americans to participate in ACP discussions with their family. Participants will be recruited from Chinese community events in Chicago. We will recruit 100 Chinese Americans who (1) are aged 18 years and older; (2) are able to read and respond to questions in English or Mandarin; and (3) self-identify as first- or second-generation Chinese Americans.

#### Procedure

We will approach potential participants at events specifically organized for Chinese Americans in Chicago. We will use a research information sheet to introduce the study to any potential participants we meet during the events. If they express interest in participating in the study, they will be encouraged to thoroughly read the information sheet and ask any questions they may have. After providing their verbal consent, they will proceed to complete the 34-item paper-and-pencil Chinese or English version of the ACP Engagement Survey, which assesses 4 behavior change constructs, including knowledge, contemplation, self-efficacy, and readiness within 4 ACP domains [43]. In addition, they will be asked to complete a demographic survey regarding their educational attainment, marital status, and employment status. They will also be invited to provide their contact information if they have any interest in participating in future research studies focusing on Chinese Americans.

#### Data Analysis Plan

Descriptive statistics, including frequencies, percentages, means, and SD, will be performed to describe the participants’ demographic characteristics and their readiness for ACP discussions.

### Aim 3

#### Setting and Sample

For aim 3, we will conduct think-aloud interviews to refine a culturally tailored, resilience-building intervention developed for and with Chinese Americans with cancer or heart disease and collect feedback on the intervention’s content. Patients and family caregivers will be recruited from 2 hospitals in Chicago, which serve at least 150 Chinese Americans with cancer or heart disease every year. We will use convenience sampling to recruit 18 pairs of patients and family caregivers (9 pairs each for cancer and heart disease; N=36) for think-aloud interviews. Patients will be eligible for the think-aloud interviews if they (1) are aged 18 years and older; (2) have a cancer or heart failure diagnosis documented in the electronic medical record; (3) are able to read and respond to questions in English or Mandarin; and (4) have a family caregiver who is responsible for the care and willing to participate in the study. Family caregivers will be eligible if they are (1) aged 18 years and older; (2) able to read and respond to questions in English or Mandarin; and (3) identified by the patient as a family caregiver. Patients who (1) have cognitive impairment per a Short Portable Mental Status Questionnaire with more than three errors; (2) have received heart transplantation or a left ventricular assist device; (3) have completed an advance directive; or (4) were born in the United States will be excluded from the study.

#### Procedure

For aim 3, after approval by the affiliated IRBs, we will request a list of potential participants who are aged 18 years or older and have cancer or heart failure diagnosis documented in the electronic medical record from each hospital. Our recruitment process will begin by sending potential patient participants an introductory email. Following the email, we will wait for a period of 2 weeks before initiating contact with them via telephone. For individuals who have only a telephone number as contact information, we will initiate a phone call to inquire about their interest in participating in the study using a telephone script. If they express interest in participating during our initial contact through email or phone call, we will use the Short Portable Mental Status Questionnaire to assess cognitive function. After the screening, we will inform eligible participants that a family caregiver responsible for their care is required in order to participate in the study. They have the option to either provide us with the contact information of their family caregiver or have their family caregiver contact us directly through email or phone. For those who share the contact information with us, we will first recruit their family caregiver by sending an email. We will then wait for 2 weeks before initiating contact via telephone. For family caregivers who only have a telephone number as their contact information, we will initiate a phone call to inquire about their interest in participating in the study and screen for their eligibility. Eligible participants will then be given a brief description of the research and asked if they wish to learn more. If they agree, a 1-hour usability test will be arranged at a time convenient for both the patient and the identified family caregiver to meet a research team member at the University of Illinois Chicago College of Nursing Behavioral Research Core Laboratory.

Prior to the usability test, participants will first be asked to review the consent form with a research team member. They will be encouraged to ask questions they may have regarding the study during this process. Once we receive their consent, we will proceed with the 1-hour usability test. Participants will be provided with a survey regarding their demographic information, a copy of the Chinese version of Five Wishes, and a resilience-building guide. While participants are reading the guide, we will encourage them to verbalize their impressions of the guide and activities. The think-aloud interviews will be audio and video recorded, along with their facial expressions and movements. Participants’ responses and expressions will be used for later refinement of the intervention. To assess the acceptability of the intervention, each participant, at the end of each interview, will be asked to complete the Theoretical Framework of Acceptability. The constructs included in the Theoretical Framework of Acceptability are affective attitude, burden, ethicality, opportunity costs, intervention coherence, perceived effectiveness, and self-efficacy, which can help us assess the acceptability of the intervention during both its development and modification [44]. On interview completion, each patient and family caregiver will receive US $50 in cash (a total of US $100 per pair) for their time and effort.

The culturally tailored, resilience-building intervention prototype will be evaluated and refined upon completion of each group of 3 patient and family caregiver pairs. Appropriate changes will be implemented based on the input and responses from the Theoretical Framework of Acceptability. The resilience-building guide will undergo a review by IRB before each subsequent round of participants reviews it for any changes made.

#### Data Analysis Plan

Data obtained through the think-aloud interviews will be transcribed verbatim; summative content analysis will be used to identify any recurrent issues. Two members of the research team will independently review the transcripts and document the occurrences of the issues. The findings will be compared until a consensus is reached between the 2 team members. All issues identified during each round of think-aloud interviews and the responses from the Theoretical Framework of Acceptability will be discussed based on the categories of the MoSCoW (Must Have, Should Have, Could Have, and Will Not Have) criteria [45]. The research team will use the MoSCoW criteria to help prioritize the necessary modifications needed, which can ensure that the intervention provides understandable and meaningful information. These modifications will contribute to a revised version of the culturally tailored, resilience-building intervention that leads to the completion of an advance directive to be optimized in the second phase of MOST.

Descriptive statistics will be calculated to assess the acceptability of the intervention through the Theoretical Framework of Acceptability. Study feasibility will be examined through (1) the number of potential patient participants who approached, screened, and consented for the aim 3 think-aloud interviews; (2) reasons for refusal to participate; (3) characteristics of the patients and family caregivers who consented versus those who refused; (4) the number of patients and family caregivers who visited the Behavioral Research Core Laboratory for the think-aloud interviews; (5) the number of tasks completed for each module during the think-aloud interviews; (6) the number of participant reminder calls completed; and (7) the facilitators and barriers to implementation and how they were addressed.

## Results

Recruitment for the qualitative study (aim 1) began in November 2023 following the IRB approval. As of October 2024, a total of 7 participants have provided consent and shared their experiences during the semistructured interviews. The response rate was initially low due to the holiday season at the end of the year, and it improved after the holidays. The snowball sampling method was not as effective as expected among the population of religious or spiritual leaders, likely due to the additional workload placed on them. Instead, contacting religious or spiritual leaders by phone using the lists of religious organizations identified on the internet in Chicago made recruitment more feasible. A preliminary qualitative analysis is currently being conducted to examine the coding guide developed from previous research.

The IRB approval for conducting surveys (aim 2) has been obtained, allowing recruitment to commence in April 2024. As of October 2024, a total of 63 participants have completed the ACP Engagement Survey. Furthermore, the process and implementation of conducting and recruiting participants for the think-aloud interviews have been approved by the affiliated IRBs, and a list of potential participants has been provided by each hospital. Recruitment may begin upon completion of preliminary qualitative analysis of aim 1.

## Discussion

This feasibility study presents a unique and innovative strategy to facilitate engagement in ACP discussions. Our use of multiple study designs allows us to anticipate that Chinese Americans diagnosed with cancer or heart disease and their family caregivers will accept the resulting prototype of the culturally tailored, resilience-building ACP intervention. Three aspects of the study are particularly innovative: (1) the intervention will include family caregivers, not just patients; (2) we will involve religious or spiritual leaders in developing the resilience-building intervention to address barriers to ACP; and (3) the study will use MOST as a guide for intervention development to optimize the effectiveness, affordability, scalability, and efficiency of the intervention. Completing the first phase of MOST prior to an RCT for intervention effectiveness can pinpoint practical intervention components in terms of the demand and resources needed for implementation [35]. This essential process will offer an opportunity to attain greater public health effects by reaching more underserved Chinese Americans through an affordable and scalable culturally tailored, resilience-building ACP intervention.

It should be noted that, in the development of a culturally tailored, resilience-building intervention, consideration should be given to participants’ acculturation [22]. Chinese Americans in Chicago tend to be low acculturated [46]; thus, the proposed intervention aims to offer support to individuals with low acculturation by providing them with exposure to ACP and methods for resolving conflicts between their health care preferences and traditional cultural values. In fact, many cultures and communities consider the topic of ACP or death to be taboo, and while the majority of the modules in the resilience guide will be tailored specifically for Chinese Americans, some modules will be useful for the general population.

Finally, when using in-person observation for the think-aloud interviews, the Hawthorne effect, which happens when participants become conscious of being observed, can occur. To prevent this, we will encourage participants to think aloud about what they see and feel about the resilience guide, and the inclusion of a survey at the end of the think-aloud interview to gather additional information can help ensure that participants’ feedback aligns with their responses during the interview. While the issues related to acculturation and in-person observations are beyond the scope of this study, they will be included in the design of future studies for the second phase of MOST.

## Appendix

Multimedia Appendix 1 Components of the resilience-building intervention to facilitate ACP discussions. ACP: advance care planning.

Multimedia Appendix 2 Peer-review report by the Chicago Chronic Condition Equity Framework (Chicago, USA).

## Abbreviations

ACP: advance care planning
IRB: institutional review board
MoSCoW: Must Have, Should Have, Could Have, and Will Not Have
MOST: multiphase optimization strategy
RCT: randomized controlled trial

## Data Availability

The datasets generated during and/or analyzed during this study are available from the corresponding author on reasonable request.
