# Supplementary material for: Applying the Multiphase Optimization Strategy for the Development of a Culturally Tailored Resilience-Building Intervention to Facilitate Advance Care Planning Discussions for Chinese Americans: Protocol for a Survey and Qualitative Study
Source: JMIR Res Protoc. 2024 Nov 26;13:e59343. doi: 10.2196/59343 (PMC11632283; doi:10.2196/59343)
Supplement: Multimedia Appendix 1 [file resprot_v13i1e59343_app1.docx]

**Table 1.** Components of the resilience-building intervention to facilitate ACP discussions. ACP: advance care planning.

| Intervention component | Targeted proximal mediator |
| --- | --- |
| Knowledge related to ACP | ACP related awareness and knowledge |
| End-of-life education | Barriers related to cultural beliefs |
| Check-ins by research staff | Concrete support |
| Family caregiver involvement | Social support |
| Resilience skills for ACP | Coping skills |
