# Supplementary material for: Applying the Multiphase Optimization Strategy for the Development of a Culturally Tailored Resilience-Building Intervention to Facilitate Advance Care Planning Discussions for Chinese Americans: Protocol for a Survey and Qualitative Study
Source: JMIR Res Protoc. 2024 Nov 26;13:e59343. doi: 10.2196/59343 (PMC11632283; doi:10.2196/59343)
Supplement: Multimedia Appendix 2 [file resprot_v13i1e59343_app2.pdf]

Primary Reviewer Comments:

Overall Impact: Extremely ambitious, much -needed, strong prevention study with negligible weaknesses. Excellent program addressing advance care planning (ACP) disparities among Chinese Americans (CHAs) in Chicago, which is a growing and hard-to-reach population. The scope of this program identifies the strengths of culturally tailored interventions for CHAs for future research and practice. The proposed research appears feasible and very likely to be achieved in the proposed time. The literature review was very thorough research. The methodology was detailed and incorporated the use of sufficient sample sizes, analysis, and evaluation tools. I find no fault with the data analysis, or conclusions. This base work, as with all work coming from this particular group, is much-needed, comprehensive, fundamentally sound, and completely justifies the authors recommendation. This program's mission aligns with the C3EN priorities and is much needed in order advance health and racial disparities in research. One size does not fit all: Overall objective is clear and concise. The literature review was very thorough research with relevant findings and necessary information. The methodology is very detailed and incorporated the use of sufficient sample sizes, and analysis. Comprehensive research tools will be utilized. No fault with the data analysis, or conclusions.

Significance Strengths

Significant of stated aims are well explained. Schematics of the plans are included.Novel approaches and options to meet community members where they are and may be effective for closing gaps and provide benefits of ACP for EOL care among CHAs:

•Embedding equity throughout tenants of the system level change model

•Culturally-specific and will impact disparities in health care and outcomes at EOL. Discusses pitfalls. Links between hypothesis,aims, methods and evaluation clear.

•Empowering community members, and faith-based organizations to help design the customized, culturally relevant program via a collaborative partnership .

• Program will build resilience that will support sustainability.

•Closing the digital divide (affordable, internet access, training, and technical support) •Increase health education to promote self-advocacy

Significance Weakness

No noted weaknesses

Investigator(s) Strengths

Author is a new investigator. The mentor is a seasoned researcher with complementary and integrated experience. The team's area's of expertise are well suited to the community -based project and they both have the appropriate education, experience and training.
Investigator(s) Weakness
There were no noted weaknesses.

Innovation Strengths
This program uses a novel approaches and methods to meet community members where they are and may be effective for closing gaps in EOL planning among CHAs.
-Presents unique strategies to include and improve engagement in CHA communities.
-RTC design is very through, rigorous and appears feasible. The use of the cohort model is advantages and and will facilitate rare exposures and will allow the study more outcomes. Clear next steps.
Innovation Weakness
There were no noted weaknesses.

Approach Strengths
Collective impact described. True CBPR approach conducting qualitative data was applied and the methodology appears feasible. Utilized at- a- glance charts for plans. A snowball sampling intervention is designed and proposed to determine the benefits of a culturally tailored EOL planning intervention for CHA in a community based setting. Proposes to filling in the gap about the impact of faith -based leaders. Inclusion and exclusion criteria described in details.
Approach Weakness
No noted weaknesses.

Community Engagement Strengths
Community involvement is set and clear. Community members and leaders are integrated from the start. The high level of community engagement will help them with making public decisions. Formed partnerships with faith-based leaders and community members. Team will meet people where they are in the context of their community to increase awareness about the program and conduct in-person recruitment. Bi-directional communication.
Community Engagement Weakness
No letter of support/collaboration was included.

Mentorship Plan Strengths
The mentorship and consultation support for the proposed work is exceptional. The researchers have been involved in much of the work in this area. The mentor's background is adequate. The regular interactions between the mentor, consultant and investigator will help improve the quality of research, productivity and successful competition for research funding and is essential to advancing health equity and reducing disparities.
Mentorship Plan Weakness
There were no noted weaknesses.

Budget comments: The budget provided by the author was written in a useful,and organized way that displays exactly how much the program has available to spend in each expense category. It was fair and equitable and included community participants to be compensated for their time and participation. Investigators in-kind.

Secondary Reviewer Comments:

Overall Impact: Important issue, highly significant, but I have questions about study population in Aim 2. Exclusion criteria of US born Chinese Americans. No reference to whether or not perspectives change from generation to generation or why US born are excluded. Also, position title where 1/3 of funding is going is a bit awkward (hourly help). ?grantsmanship (program manager is most appropriate).

Significance Strengths
Lower rates of advanced care planning in Chinese American community due to cultural factors.
Significance Weakness
no differentiation on degree of acculturation (new immigrants vs. later generations) and differences in end of life discussions

Investigator(s) Strengths
track record in communications/resilience applications in breast cancer,
Investigator(s) Weakness
grantsmanship: description of "hourly help" in budget description

Innovation Strengths
incorporation of community religious leaders and patient/family clusters to discuss barriers to end of life discussions

Innovation Weakness
common approach

Approach Strengths
use of resilience framework, experience of investigators in applying framework to same population with breast cancer
Approach Weakness
limited recruitment from Rush University Medical Center patients. lacking description of why no US born Chinese Americans are included in the study.

Community Engagement Strengths
some outreach to pastors in Aim 1
Community Engagement Weakness
no specificity on outreach to community religious leaders, i.e. identification of organizations

Mentorship Plan Strengths
two mentors: methodology and cultural competence
Mentorship Plan Weakness
None

Budget Comments: appropriate, though description of hourly help is somewhat confusing.
